# Treated well but feeling poorly: examining supportive care devices in oncology practice

**DOI:** 10.1093/oncolo/oyaf116

**Published:** 2025-05-27

**Authors:** Quan H Phung, Arjun Gupta, Gabrielle B Rocque, S M Qasim Hussaini

**Affiliations:** Masonic Cancer Center, University of Minnesota, Minneapolis, MN 55455, United States; Masonic Cancer Center, University of Minnesota, Minneapolis, MN 55455, United States; University of Alabama at Birmingham Heersink School of Medicine, Birmingham, AL 35233, United States; O’Neal Comprehensive Cancer Center, University of Alabama at Birmingham, Birmingham, AL 35243, United States; University of Alabama at Birmingham Heersink School of Medicine, Birmingham, AL 35233, United States; O’Neal Comprehensive Cancer Center, University of Alabama at Birmingham, Birmingham, AL 35243, United States

**Keywords:** supportive care, reimbursement, treatment toxicity, advocacy, policy

As the array of cancer therapeutics has increased, so too has the range of potential adverse effects from treatment. Although symptoms vary greatly among different types of cancers and treatment regimens, most patients undergoing systemic therapy experience some form of acute or chronic side effects.^1^ Supportive care is a growing field that addresses these side effects. The Multinational Association of Supportive Care in Cancer (MASCC) defines supportive care to include prevention and management of the adverse effects of cancer and its treatment.^2^ Multimodal management should incorporate resources such as supportive devices, procedures, or services. In this article, we discuss the role of supportive care devices in oncologic care, barriers to appropriate uptake and evidence generation for these devices, and potential solutions that may help structure future improvements. We begin with a focus on supportive care device-related developments that target treatment side effects.

## Supportive care innovations with devices

### Alopecia

Scalp cooling devices can support patients with chemotherapy-induced alopecia which is a common adverse effect impacting approximately 65% of patients receiving cytotoxic therapies.^3^ Scalp cooling devices function by causing local vasoconstriction and decreasing chemotherapy delivery to hair follicles.^4^ Two systematic reviews and meta-analyses showed approximately a 40% decrease in the risk of chemotherapy-induced alopecia for patients undergoing breast cancer treatment who used a scalp cooling device.^5,6^ Similarly, a systematic review involving patients with gynecologic cancer found scalp cooling devices reduced chemotherapy-induced alopecia among patients who received taxane-based chemotherapy.^7^

### Lymphedema

Another common concern for patients with cancer is lymphedema.^8^ This is especially prevalent among individuals treated for breast cancer, since approximately 1 out of 5 of these patients will experience chronic upper limb, trunk, or breast lymphadenopathy.^9^ Multiple studies have shown that compression stockings, which function to reduce swelling by applying pressure to affected body parts and preventing fluid accumulation, help to reduce lymphedema.^10,11^

### Neuropathy

Cooling devices also offer a promising avenue to treat peripheral neuropathy. Cold gloves and socks have been developed to reduce the potentially debilitating effects of chemotherapy-induced peripheral neuropathy (CIPN). A literature review of regional cooling devices showed that 4 out of the 6 studies examined demonstrated significant benefits in reducing the severity of CIPN.^12^ Additionally, a meta-analysis showed that prophylactic cryotherapy reduced taxane-induced peripheral neuropathy.^13^ Oral cryotherapy (ice chips), though not a device, function in a similar manner to decrease oral thermal hyperalgesia and mucositis.^14,15^ However, for these cooling interventions, there is no established consensus on intervention parameters such as temperature, duration, or frequency of use. In the absence of formal guidelines some patient advocate groups on social media have shared “icing protocols” based on patient experiences.^16^ While these recommendations are well-intentioned and can be helpful, they are largely based on anecdotal evidence and not translated into standard clinical practice, so there remains a need for high-quality clinical trials.

### Nausea

Several devices have been developed to try to reduce nausea, a pervasive side effect of chemotherapy. A systematic review involving aromatherapy inhalers for chemotherapy-induced nausea and vomiting (CINV) found that most of the studies reviewed (7 of 9) showed a positive benefit in reducing CINV for adults.^17^ While multiple studies showed a benefit of aromatherapy in reducing nausea, the type of essential oil, scent, number of drops, and method of administration varied significantly and there remains a need for additional randomized clinical trials.^17,18^ Anti-nausea wristbands, which typically function to apply increased pressure to the wrist and have been used for motion sickness and pregnancy-related nausea, have not been proven to be effective in improving CINV.^19,20^ Transcutaneous electrical nerve stimulation (TENS) utilizes low-intensity electrical pulses in an effort to lower pain or nausea. Although there is data that this could potentially reduce the severity of nausea, the overall evidence has been inconclusive, with some studies showing efficacy while others failing to show a significant difference from placebo.^21,22^ Relatedly, transcutaneous electrical acupoint stimulation (TEAS) combines elements of TENS with acupuncture to deliver low-intensity electrical pulses to acupuncture points. TEAS has not been proven to be efficacious in reducing the overall incidence of CINV, though it may help reduce the severity of nausea and the incidence of anorexia.^23,24^

A common theme is that the lack of high-quality studies and significant heterogeneity between studies limits definitive conclusions. As the evidence remains imprecise primarily derived from small single-center randomized trials, there needs to be a concerted effort to develop better higher quality multi-center trials with large sample sizes. Until then, some devices should only be used under clinical trials or appropriately designed real-world implementation programs.

In **Table 1**, we provide a list of common aforementioned supportive care interventions, evidence surrounding their care, nature of support, current insurance coverage, and cost-sharing to the patient.

**Table 1.** Overview of selected supportive care devices.

Intervention	Indication	Prevalence	Evidence	Insurance coverage	Cost to patient	Need for support infrastructure	Nature of support
Compression stockings	Lymphedema	High	Robust	Yes	$	Low	Call to pharmacy; patient teaching
Cold gloves/socks	Neuropathy	Low	Limited	Rarely	$$	High	Provider education; coolers/freezers; frozen gloves/socks
Scalp cooling	Alopecia	Low	Limited	Preliminary approval	$$$	High	Provider education; licensed device
TENS and TEAS	Nausea	Low	Limited	Rarely	$$	High	Provider education; licensed device; patient teaching

^*^Prevalence refers to the general likelihood that patients will utilize intervention at some point during their oncologic care. *Evidence: “Robust” refers to an established benefit or researched through randomized controlled trials (RCT; ≥5 studies). “Limited” refers to a benefit that is not well-established or based on non-randomized studies or expert opinion. TENS: transcutaneous electrical nerve stimulation, TEAS: transcutaneous electrical acupoint stimulation.

## Barriers to uptake of supportive care interventions

**Figure 1.**
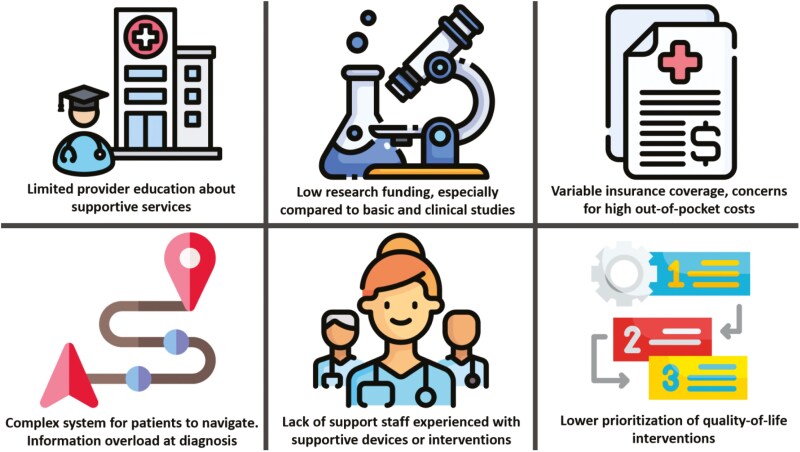
Common barriers to supportive care interventions.

Despite such advances in supportive care, there remain concerning gaps in preventing and treating common toxicities. In contrast to the well-developed research and regulatory infrastructure surrounding the approval and uptake of drugs, the development and implementation of supportive care devices face considerable challenges with resulting barriers (**Figure 1**). We often also hold supportive care interventions to a higher standard for return on investment than new cancer-directed treatments.^25^ Limited research can lead to a cycle of poor adoption and real-world use of supportive devices, which further limits future research investment. It is worth noting that many of the barriers below not only prevent the uptake of supportive care interventions but also the generation of high-quality evidence that is still needed to support their use.

### Clinician knowledge gaps

One limitation to the use of supportive care devices is a lack of clinician education and awareness about supportive interventions. Medical education for oncologists tends to focus on cancer-directed treatment. We are taught about dose adjustments and prescribing additional medications to help address symptoms but rarely receive formal education about alternative supportive measures. As a result, clinicians often lean on our patients to share their experiences with these devices. However, patients face a confusing and crowded array of supportive products, some of which have no clear benefit.

### Logistical and staffing hurdles

Limited knowledge about supportive devices is compounded by a lack of support staff to assist patients and providers in navigating the process of requesting, obtaining, and implementing these supportive measures. For instance, some interventions, such as scalp cooling require patients to be carefully fitted by a trained technician. However, due to unclear guidance on specific devices and usage parameters, patients often resort to online retailers, such as Amazon, or create their own make-shift solutions. Under such a logistical burden, patients often feel overloaded and concerned that focusing on supportive care may delay their cancer treatment.^26,27^

### Cost and insurance coverage

Concerns about out-of-pocket costs and lack of insurance coverage are major deterrents to seeking supportive care. These devices are inconsistently covered by insurers and when there is coverage, patients may still face difficulties such as narrow indications for which plans will cover these devices or requiring patients to try certain medications or other interventions before these devices could be considered. Without coverage, patients face hefty, often unaffordable prices. For example, for those undergoing treatment for breast cancer, the estimated out-of-pocket cost is $2000-2200 for scalp cooling through a course of chemotherapy.^28^ The Centers for Medicare & Medicaid Services (CMS) recently reassigned a CPT code to the Paxman scalp cooling system with a national average Medicare payment of $1850.50.^29^ Further, the American Medical Association (AMA) introduced new Category I CPT codes for scalp cooling services, which will take effect starting in 2026.^30^ While coverage for scalp cooling is improving, this trend is not universal. As a result, patients who already have greater financial means may be more likely to receive supportive measures, which risk exacerbating existing socioeconomic disparities in cancer care.^31^

For patients, the downstream effects of these barriers are real, and can go unnoticed during clinic. In the absence of coverage, we have encountered firsthand accounts of distraught patients financially prioritizing untested online treatments for managing side effects over paying for basic needs such as secure and stable housing. Many open new credit lines, resulting in further financial toxicity and pronounced psychological distress. One patient we encountered had trained to be a violinist from an early age. Unfortunately, he developed significant peripheral neuropathy and he could no longer play the violin after curative treatment, a tragic twist of fate for what should have been a celebratory moment. These types of downstream consequences are rarely appreciated and deserve our attention.

## Empowering action—building evidence, facilitating appropriate use, and policy advocacy

### Prioritizing education and high-quality research

Future actions to build evidence and, where supported, implement supportive care interventions may be approached at the patient-provider and policy levels. At the patient-provider level, it begins with clinician education about the current evidence limitations and ongoing trials. When clinicians are informed about supportive care devices, it will allow them to better provide appropriate counseling about the risk of treatment-related side effects, what interventions would be available to treat symptoms, and steer patients in the direction of new research protocols or implementation programs.

In addition to being broadly informed about these interventions, there is a need for further high-quality research on supportive care devices, to better characterize which devices can meaningfully improve symptoms and quality of life. Interested providers have a unique opportunity to collaborate with engineers and device manufacturers to facilitate the combination of the technical skills needed for device development with the clinical experience necessary to identify specific patient needs. It is worth first emphasizing that the adoption of supportive care interventions may be an entirely rational response to uncertain benefits. To advocate broader deployment, there will need to be higher-quality multi-center randomized evidence looking at patient-centered outcomes alongside real-world evidence that can track device exposure and quality-of-life metrics.

### Policy and reimbursement landscape

At a broader level, policies that promote the use of and coverage of supportive measures can be an effective tool to encourage their adoption into medical practice. For example, The Women’s Health and Cancer Rights Act of 1998 requires plans that cover mastectomies to also cover breast reconstructive surgery and physical complications of mastectomy such as lymphedema.^32^ Thus, this coverage extends to devices like lymphedema sleeves and elastic bandages or wraps, contributing to improved access for patients. Relatedly, the Lymphedema Treatment Act is a federal law that went into effect in 2024, which provides Medicare coverage of compression lymphedema items.^33^ Unique models of reimbursement may also be pursued including conditional payment policies such as CMS’ Coverage with Evidence Development program as well as reimbursements implemented through a time-limited technology add-on payment that could be used to help spur additional data collection before widespread adoption of an uncertain technology. While influencing policy can be challenging for medical providers, these policy initiatives have a broad impact on patient care and are worth collectively pursuing.

## Conclusions

As oncologists, we treat our patients with state-of-the-art treatments that undeniably come with their fair share of grade 3 or 4 toxicities. We are encouraged by the many advances in pharmaceuticals to treat cancer, however, we do not want to lose sight of the importance of alleviating symptom burden and quality of life. As a previous patient at our training institution, now a patient advocate, poignantly remarked to us that, as doctors we did a formidable job treating them, but fell short of making them actually feel better. Just as we introduce a growing array of new advances and treatment-related adverse reactions, oncology as a field has a responsibility to foster the examination, development, and coverage of supportive care interventions in our patients.

## Data Availability

No new data were generated or analysed in support of this research.
